# Assessing the validity of a cross-platform retinal image segmentation tool in normal and diseased retina

**DOI:** 10.1038/s41598-021-01105-9

**Published:** 2021-11-08

**Authors:** Varsha Alex, Tahmineh Motevasseli, William R. Freeman, Jefy A. Jayamon, Dirk-Uwe G. Bartsch, Shyamanga Borooah

**Affiliations:** 1 Jacobs Retina Center, Shiley Eye Institute, University of California San Diego, 9415 Campus Point Drive, La Jolla, CA 92093 USA; 2 Qualcomm Technologies Inc, San Diego, CA USA

**Keywords:** Outcomes research, Translational research

## Abstract

Comparing automated retinal layer segmentation using proprietary software (Heidelberg Spectralis HRA + OCT) and cross-platform Optical Coherence Tomography (OCT) segmentation software (Orion). Image segmentations of normal and diseased (iAMD, DME) eyes were performed using both softwares and then compared to the ‘gold standard’ of manual segmentation. A qualitative assessment and quantitative (layer volume) comparison of segmentations were performed. Segmented images from the two softwares were graded by two masked graders and in cases with difference, a senior retina specialist made a final independent decisive grading. Cross-platform software was significantly better than the proprietary software in the segmentation of NFL and INL layers in Normal eyes. It generated significantly better segmentation only for NFL in iAMD and for INL and OPL layers in DME eyes. In normal eyes, all retinal layer volumes calculated by the two softwares were moderate-strongly correlated except OUTLY. In iAMD eyes, GCIPL, INL, ONL, INLY, TRV layer volumes were moderate-strongly correlated between softwares. In eyes with DME, all layer volume values were moderate-strongly correlated between softwares. Cross-platform software can be used reliably in research settings to study the retinal layers as it compares well against manual segmentation and the commonly used proprietary software for both normal and diseased eyes.

## Introduction

Since its invention in 1991, Optical Coherence Tomography (OCT) has greatly assisted both ophthalmic clinical and research imaging^1^. Spectral Domain Optical Coherence Tomography (SD-OCT) systems are now able to rapidly capture high-resolution, three-dimensional (3D) volume scans of the retina for identifying, monitoring, and quantitatively assessing various pathologic conditions of the macula^1^. Alongside the improvement in scanning speed, SD-OCT is capable of visualizing the retina and its sublayers at a greater resolution. The use of SD-OCT has increased rapidly during the last two decades.

Manual segmentation of retinal layers from SD-OCT images is time consuming^2^. Automated segmentation potentially allows for rapid, accurate and repeatable delineation of individual retinal layers assisting the investigation of retinal diseases^2^. While automated retinal segmentation algorithms have traditionally performed well in normal retina to segment major retinal landmarks, there is a relative lack of data for automated segmentation of inner retinal layers in pathology^3,4^.

The Heidelberg HRA + OCT system is now used globally for retinal studies in both the clinic and research settings. The proprietary software included with the Heidelberg system has been continually updated and has allowed intra retinal segmentation from version 6^1^. However, the software had limitations which made its use difficult. For example, currently the Spectralis software does not perform choroido-scleral interface segmentation automatically, in volume scan OCTs. This requires manual segmentation of individual B-scans. Additionally, although commercial OCT devices have on-board proprietary segmentation software which are fast and designed to give reliable values for interpretation by clinicians, the definition of the retinal boundaries varies between manufacturers, and this makes quantitative retinal thickness comparisons difficult. Proprietary software is almost always limited to images captured by the parent device and cannot be applied to images from other OCT devices^4^. The algorithms are normally not accessible due to their proprietary nature, forcing the development of independent custom-built software. The initial iteration of Heidelberg proprietary segmentation software was considered inaccurate for segmenting retinal pathology^5^. However, the software has now been updated.

Modern cross-platform softwares offer to overcome some of these drawbacks^6^. One cross-platform system, accessible using a subscription model, has been developed by Voxeleron (Voxeleron LLC, Pleasanton, CA, USA). Their cross- platform software (Orion) is reported to provide device independent eight-layer retinal segmentation. It has recently been used for a number of retinal disease studies including longitudinal measurement of retinal layer volumes in AMD^7^, layer segmentation in retinitis pigmentosa^8^, glaucoma and retinal manifestations of neurological disease^9–11^. This software measures retinal layer volumes with distinct boundaries which include the Nerve Fiber Layer (NFL), Ganglion Cell-Inner Plexiform Layer (GCIPL), Inner Nuclear Layer (INL), Outer Plexiform Layer (OPL), Outer Nuclear Layer (ONL), Photoreceptors (PR) and Retinal Pigment Epithelium–Bruch’s Membrane complex (RPE-BM). Additionally, the software can rapidly add new layer segmentations, such as the choroido-scleral interface with a semi-automated input. It measures the retinal volumes in the central macular area (6 mm diameter) automatically centered on the fovea, thereby supporting longitudinal analysis. This software has already proven to be reliable in the retinal layer segmentations captured using the Topcon 3D OCT-2000 imaging system^12^. However, there has been little study of retinal segmentation using this cross-platform software on images captured using Heidelberg HRA + OCT systems.

In this study, we compare retinal layer segmentation performed by the proprietary software of the Heidelberg system and the cross-platform software developed by Voxeleron using scans from normal eyes and eyes with pathology. First, we qualitatively assess how well both the softwares can segment retina compared to the gold standard manual segmentation and then try to understand how retinal segmentation is different between the software by quantitatively comparing differences in measurements of retinal layer volumes between the softwares in normal and diseased retina.

## Methods

Institutional Review Board (IRB) approval was acquired from the University of California San Diego for the review and analysis of patients’ data. Patient’s informed consent was obtained as per institutional protocol and all data and images were anonymized for patient’s safety. This retrospective cross-sectional study was conducted according to the principles of the Declaration of Helsinki. The study complied with the Health Insurance Portability and Accountability Act of 1996. List of patients who presented to Jacobs Retina Center (JRC), University of California San Diego between 1st January 2019 and 31st December 2020 with diagnosis of Normal eyes, eyes with intermediate dry Age-related Macular Degeneration (iAMD), representing an outer retinal pathology commonly seen in the clinical setting, and diabetic retinopathy with Diabetic Macular Edema (DME), representing an inner retinal pathology commonly seen in clinic, were identified from the retinal imaging report database. Pathology was confirmed by a senior retina specialist (WRF). iAMD was confirmed if patients had at least one drusen > 125 µm in the eyes being classified as iAMD.

Images were captured using a standard protocol for imaging all patients attending the Jacobs Retina Center involving forty-nine-line volume scans. The Spectralis SD‐OCT with the HRA + OCT protocol was used, and in each eye, a macular area (6 × 6 mm^2^) cube centered on the fovea was scanned with an Automated Real Time (ART) of 16 as part of the routine protocol. Retinal layer segmentations of the ETDRS (Early Treatment Diabetic Retinopathy Study) zone were performed using two different softwares. The first software was the proprietary software (Heidelberg Eye Explorer, Heidelberg Engineering GmbH, Heidelberg, Germany, version 1.10.4.0, running HRA / Spectralis Viewing Module 6.15.7.0) provided along with Heidelberg Spectralis HRA + OCT. The second software was the cross-platform automated OCT layer segmentation software (Orion, Voxeleron LLC, Pleasanton, CA, USA, version 3.0.0). The cross-platform software was installed on a PC running Windows 7 after downloading the software from the Voxeleron server. The PC has multiple monitor support and uses two identical Asus, 24” LCD displays (Model—VS248H-P). OCT images (49-line volume scans) of the eyes captured using the Spectralis SD-OCT machine were first exported as E2E files and then imported into the cross-platform software (Orion) for segmentation. The proprietary software could perform the image segmentation directly without the need for export.

**Figure 1:**
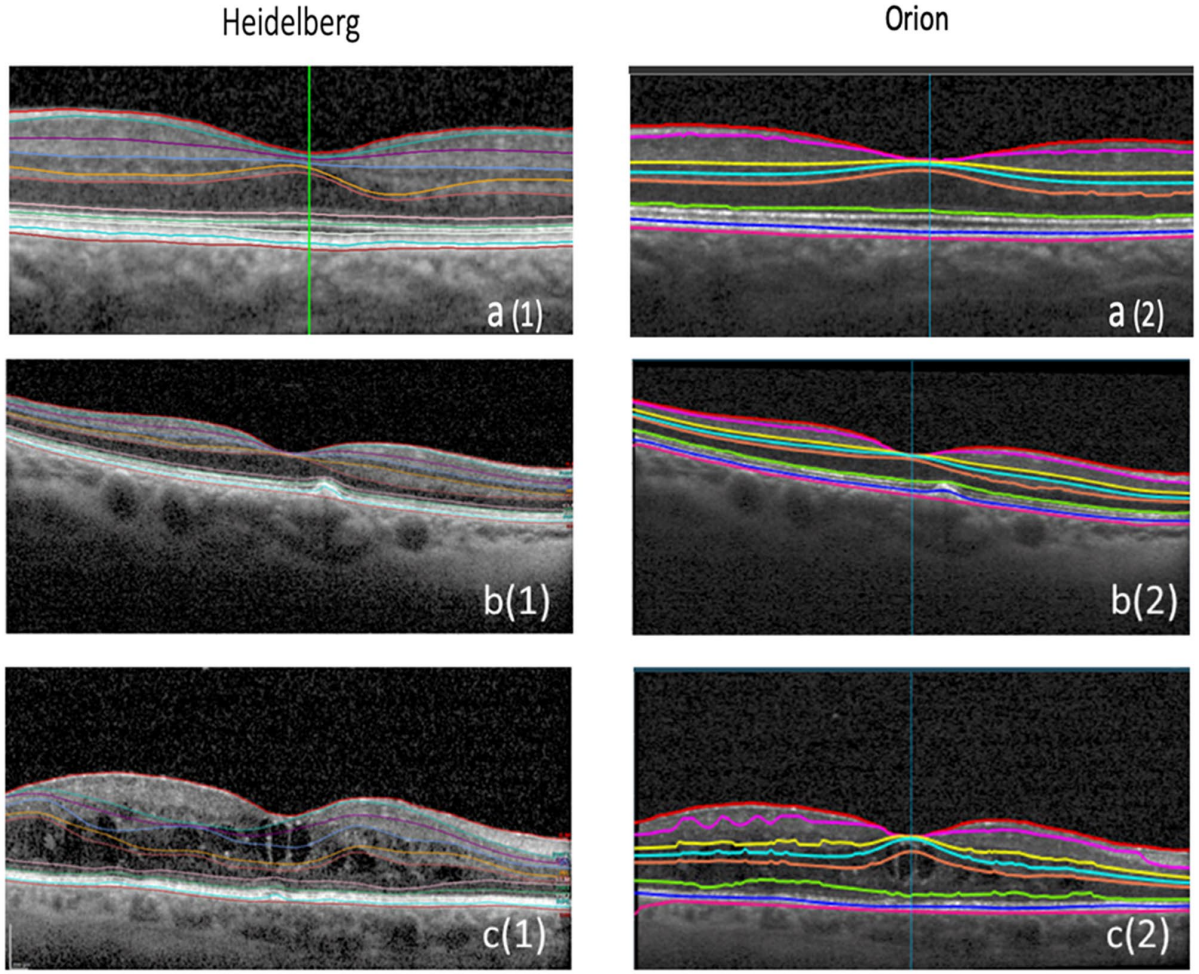
Representative images of retinal segmentation of Normal, Intermediate dry AMD (iAMD) and DME eyes using proprietary software (Heidelberg Spectralis) and automated OCT layer segmentation cross-platform software (Orion). (**a1**) Normal eyes using Proprietary software, (**a**(2)) Normal eyes using Cross platform software, (**b**(1)) iAMD eyes using proprietary software, (**b**(**2**)) iAMD eyes using cross platform software, (**c**(1)) DME eyes using Proprietary software, (**c**(2)) DME eyes using Cross platform software.

Images from 45 Normal eyes, 33 iAMD eyes and 30 eyes with DME were used for the assessment, the representative images of which are shown in Fig. 1. There were 22 patients with both eyes (44 eyes) analyzed in the Normal group, 10 patients with both eyes (20 eyes) analyzed in the iAMD group and 9 patients with both eyes (18 eyes) analyzed in the DME group. Exclusion criteria included images which were incomplete or unclear in one or more B scans and images with values more than 3 sigma from mean population values. Additionally, Enhanced Depth Imaging (EDI) volume scans in Proprietary software were also excluded from the study because they could not be segmented and exported as raw data (E2E files) to the cross-platform software. In Normal patients, there were a total of fifty-nine eyes to start with of which fourteen eyes were excluded as outliers because their segmented layer volume data was beyond 3-sigma from mean population values. Similarly, out of the 42 iAMD eyes initially, nine images were excluded due to the incomplete layer volume data in B scans. Out of the 37 DME eyes, three eyes were excluded because they had only EDI scans and four eyes with Total segmented Retinal layer Volume (TRV) data beyond 3 sigma from mean population values were discarded as outliers. To conclude, after the exclusion of 14 eyes from Normal, 9 eyes from iAMD and 7 eyes from DME, the remaining 45 Normal eyes, 33 iAMD eyes and 30 DME eyes were included in the study making a total of 108 eyes.

The segmented retinal layer volume was measured in mm^3^ and the thickness in microns. Quantitative comparisons were made between the different retinal layer volumes measured with the proprietary software and the cross-platform software. Volumes of NFL, GCL, IPL, INL, OPL, ONL, INLY (Inner retinal layer in toto) and OUTLY (Outer retinal layer in toto) were obtained in the Proprietary software. The INLY was defined as the volume lying between the ILM and ELM (interior border) and OUTLY was defined as the volume lying between the ELM (outer border) and the RPE-Bruch’s membrane complex. NFL, GCL_IPL, INL, OPL, ONL, PR and RPE_BRUCHS layer volumes in mm^3^ were obtained from the output (csv) files in the cross-platform software. The GCL and IPL layers in the cross-platform software were considered a single layer and hence the two-layer volumes in the proprietary software were added to match the cross-platform software. Similarly, PR and RPE_BRUCHS layer volumes were considered a single layer in the cross-platform software. Total retinal layer volumes (TRV) were also obtained from both the proprietary software and the cross-platform software.

For qualitative analyses of the images, the degree of segmentation error in the proprietary software and the cross- platform software were graded as good, mild, moderate, or severe by visual verification. This was done using reference images (Supplementary Figs. S1, S2 and S3) by two masked retina fellows, VA and TM, who compared the segmentation to where the segmentation was expected to be. A total of 8 surfaces (per one foveal B-scan) for 108 eyes, adding up to 864 surfaces were checked and graded by each fellow. The qualitative grading was based on the difference from the visual verification of images. For the visual verification of the segmentation, the grader looks at the automated segmented layer boundaries and qualitatively estimates the accuracy of the segmentation. Intergrader agreement was calculated using a Kappa statistic. In instances where there was disagreement between the two graders, a third masked grader/senior retinal specialist (SB) made a final decisive grading. The number of surfaces (percentage of times) in proprietary software where the third expert grader intervened to break the tie in Normal eyes was 24%, in AMD eyes was 44% and in DME eyes was 47%. The number of surfaces (percentage of times) in the cross-platform where the third senior grader had to intervene in Normal eyes was 10%, in AMD eyes was 26% and in DME eyes was 42%. Wilcoxon analysis was used to compare the automated segmentation gradings between the two softwares.

To further strengthen our study, we performed the gold standard ‘true’ manual retinal layer segmentation in a subset of eyes from each patient group, as previously done by Rossant et al.^13^ and Neimeiier et al.^14^. With the help of the same two masked graders (VA & TM), we manually outlined the retinal layers in the B-scan images. A total of 30 images, 10 each of Normal, iAMD and DME eyes were segmented. Graders labeled 8 retinal surfaces in these representative images. Thus, a total of 240 surfaces (8 surfaces per foveal B-scan) were checked and manually outlined by each grader in the dataset. After ‘true’ manual segmentation, the images were then compared to the automated segmentation gradings of the proprietary and cross-platform softwares for a complete qualitative analysis.

One of the initial limitations of our study was that although an attempt was made to mask the graders by using the same section of images to avoid software identifiable features, the proprietary and the cross-platform retinal segmentation appeared slightly different and as a result the graders may have identified which software was used and the differences in segmentation line thicknesses may have biased the grading. In order to address this important concern, on our request, the cross-platform software manufacturer provided a new software build with segmentation which matched that of the proprietary software. Secondly, to reduce potential bias due to retinal layer segmentation line color, the color of the retinal layer lines of the cross-platform software was changed to match that of the proprietary software.

In addition, during the course of our research, the viewing module of the proprietary software had been updated once further. The Heidelberg Eye Explorer software version used was 1.10.4.0. While there had been no change in the software version of the main Heidelberg Eye Explorer, to ensure that the segmentation had not changed with the viewing module update, a sample set of 10 images of Normal, iAMD and DME eyes each of which were previously segmented with the old viewing module (6.12.7.0) version of the proprietary software were then segmented with the new Spectralis viewing module (6.15.7.0) version. These were later quantitatively and qualitatively analyzed.

For statistical analyses, Microsoft Excel 2016, and statistical software R (3.4.2, September 2017)^15^ were used. A p value of < 0.05 was taken as indicating statistical significance. The correlation strength for the layer volumes was classified based on the Pearson correlation coefficient values into weakly positive (Pearson coefficient, r < 0.4), moderately positive (Pearson coefficient, 0.4 ≤ r < 0.7) and strongly positive (Pearson coefficient, r ≥ 0.7).

## Results

### Retinal layer segmentation in normal eyes

In order to investigate how well the two software systems were able to segment images generated by the Heidelberg HRA + OCT machine, we first compared the automated retinal layer segmentations of images from Normal eyes with that of visual verification. A total of forty-five eyes were analyzed. In Normal eyes, retinal segmentation using proprietary software found, 82% to have good, 17% mild and 0.9% to have moderate segmentation error (Table 1), while the cross-platform software was found to have 97% good, 2% mild and 0.4% moderate segmentation error compared to visual verification. A qualitative comparison performed using Wilcoxon test (Table 2) found that the cross-platform software was significantly better than the proprietary software in the segmentation of NFL and INL layers (p < 0.05) (Table 2). For all other layers, there was no significant difference between the two softwares. In Normal eyes, the NFL demonstrated the least agreement in the qualitative analysis (Supplementary Table S1). Using the proprietary software, the intergrader agreement for all layers was 76%, with a kappa statistic of 0.32. Using the cross-platform software, the intergrader agreement for all layers was 90% with a kappa statistic of 0.38 (Supplementary Table S2). The test–retest kappa average for Normal eyes was 0.91.

**Table 1:** Percentage of segmentation error gradings in normal eyes, in iAMD eyes and in eyes with DME using the proprietary (HB) and the cross-platform (OR) softwares.

Normal eyes	Good	Good	Mild	Mild	Moderate	Moderate	Severe	Severe
	HB	OR	HB	OR	HB	OR	HB	OR
Percentage	81.78	97.33	17.33	2.22	0.89	0.44	0.00	0.00

iAMD eyes	Good	Good	Mild	Mild	Moderate	Moderate	Severe	Severe
	HB	OR	HB	OR	HB	OR	HB	OR
Percentage	70.91	79.39	24.24	15.76	4.85	4.85	0.00	0.00

DME eyes	Good	Good	Mild	Mild	Moderate	Moderate	Severe	Severe
	HB	OR	HB	OR	HB	OR	HB	OR
Percentage	41.33	61.33	25.33	26.00	19.33	12.67	14.00	0.00

**Table 2:** Wilcoxon test comparing different layers in proprietary and cross-platform softwares.

Qualitative comparison of grading of retinal segmentation using cross-platform (Orion) and proprietary (Heidelberg) softwares to visual verification, in normal, intermediate dry AMD and diabetic macular edema eyes			
	Mean ± SD [Proprietary software (Heidelberg)]	Mean ± SD [Cross platform software (Orion)]	p-value
**Normal eyes (N = 45)**			
NFL	1.42 ± 0.50	1.09 ± 0.36	0.001
GCIPL	1.16 ± 0.37	1.02 ± 0.15	0.07
INL	1.22 ± 0.47	1.00 ± 0.00	0.004
OPL	1.11 ± 0.38	1.00 ± 0.00	0.13
ONL	1.04 ± 0.21	1.04 ± 0.21	1.00
NFL	1.39 ± 0.56	1.15 ± 0.44	0.04
**Intermediate dry AMD eyes (N = 33)**			
GCIPL	1.21 ± 0.55	1.18 ± 0.53	0.88
INL	1.30 ± 0.53	1.15 ± 0.44	0.18
OPL	1.33 ± 0.60	1.21 ± 0.49	0.41
ONL	1.46 ± 0.62	1.58 ± 0.66	0.49
NFL	2.00 ± 1.08	1.63 ± 0.81	0.11
**Diabetic Macular Edema eyes (N = 30)**			
GCIPL	1.83 ± 1.09	1.43 ± 0.68	0.05
INL	2.43 ± 0.97	1.27 ± 0.64	0.00
OPL	2.20 ± 1.19	1.53 ± 0.68	0.003
ONL	1.83 ± 1.02	1.70 ± 0.70	0.49

Segmentation quality grading scheme: 1 = Good, 2 = Mild, 3 = Moderate, 4 = Severe.

**Figure 2:**
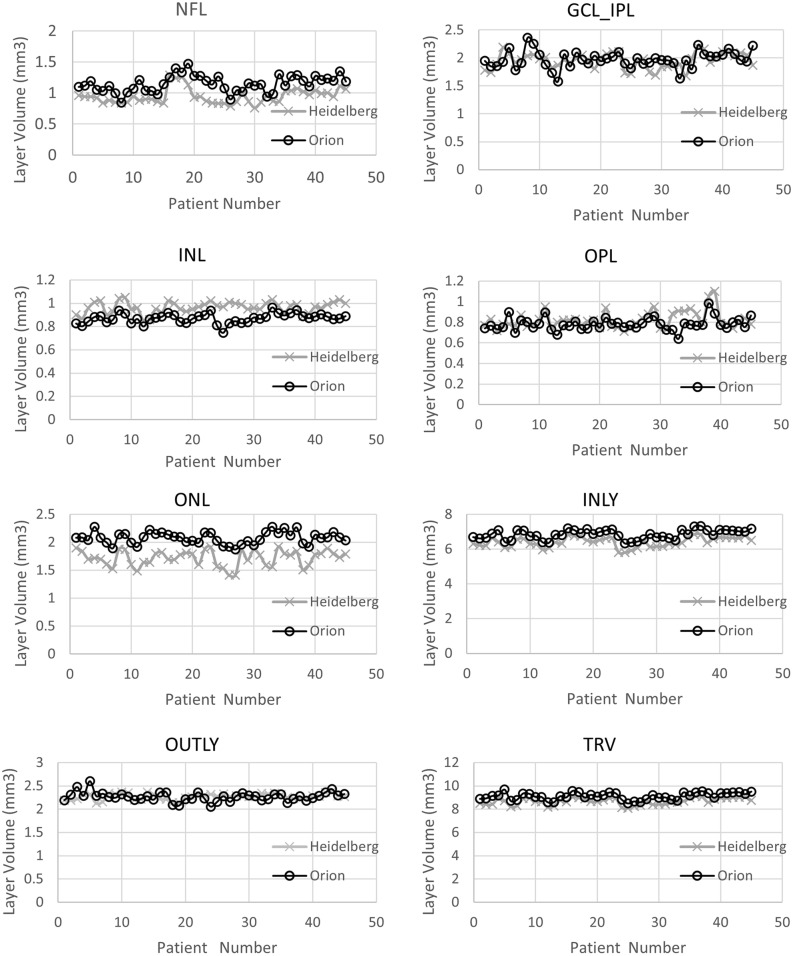
Comparison of retinal layer volumes between proprietary and cross-platform softwares in normal eyes.

To better understand where the differences between the software existed in retinal segmentation in Normal eyes, we performed a quantitative comparison of the proprietary software and the cross-platform software retinal layer segmentation. This analysis showed that the NFL, ONL, INLY and TRV layers had strongly positive correlation (r ≥ 0.7), GCL_IPL, INL, OPL layers had a moderately positive correlation (0.4 ≤ r < 0.7) and only OUTLY had a weakly positive correlation (r < 0.4) (Table 3). Paired t-test comparisons showed that there was no significant difference between the proprietary software and the cross-platform software in the GCL_IPL and OUTLY volumes. However, there was a significant difference in all other layers (p ≤ 0.05) (Table 3), (Fig. 2). In summary, although there was a general correlation between the softwares in retinal segmentation, in Normal eyes there were significant differences in measurement of layer volumes suggesting that different landmarks were used by the softwares for segmentation.

**Table 3:** Layer volume data in normal eyes from the proprietary and the cross-platform softwares.

Vol. of the retinal layers in ETDRS zone (Normal eyes)				
	Mean ± SD [Proprietary software (Heidelberg)]	Mean ± SD [Cross platform software (Orion)]	Pearson correlation	p value (paired t-test)
NFL	0.95 ± 0.12	1.14 ± 0.13	0.74	< 0.001
GCL_IPL	1.95 ± 0.14	1.97 ± 0.15	0.62	0.25
INL	0.97 ± 0.05	0.87 ± 0.04	0.40	< 0.001
OPL	0.82 ± 0.08	0.78 ± 0.06	0.67	< 0.001
ONL	1.72 ± 0.14	2.08 ± 0.11	0.74	< 0.001
INLY	6.41 ± 0.28	6.85 ± 0.28	0.94	< 0.001
OUTLY	2.27 ± 0.07	2.27 ± 0.10	0.29	0.68
TRV	8.68 ± 0.29	9.12 ± 0.31	0.95	< 0.001

### Segmentation in eyes with intermediate dry AMD

Having compared retinal layer segmentation between the softwares in Normal eyes, we then compared layer segmentation in eyes with pathology. iAMD was chosen as a prototypical disease for outer retinal pathology as it mainly affects the outer retina leaving the inner retina predominantly unaffected^4^. Thirty-three eyes with iAMD were analyzed. Out of 33 eyes, 71% were reported as good, 24% were reported mild and 4.9% were reported as moderate segmentation error by proprietary software whereas the cross-platform reported 79% segmentation as good, 15.8% as mild and 4.9% as moderate segmentation error compared to visual verification (Table 1). The qualitative analysis comparison performed using Wilcoxon test (Table 2) showed that the cross-platform segmented retina in iAMD eyes is significantly better than the proprietary software segmentation only for the NFL layer (p ≤ 0.05). For all other layers, the comparison did not find significant differences between the softwares. When comparing proprietary and the cross-platform softwares with visual grading in iAMD eyes, the average level of agreement between proprietary software and the cross-platform software for all layers was 73% and the kappa statistic average of all layers was 0.36. In iAMD eyes, ONL was the layer with most discrepancy in the qualitative analysis (Supplementary Table S1). Using proprietary software, the intergrader agreement for all layers was 56% and the kappa statistic average of all layers was 0.29. For the cross-platform images, the intergrader agreement for all layers was 74% and the kappa statistic average of all layers was 0.51 (Supplementary Table S2). The test–retest kappa average for eyes with intermediate dry AMD was 0.87.

**Figure 3:**
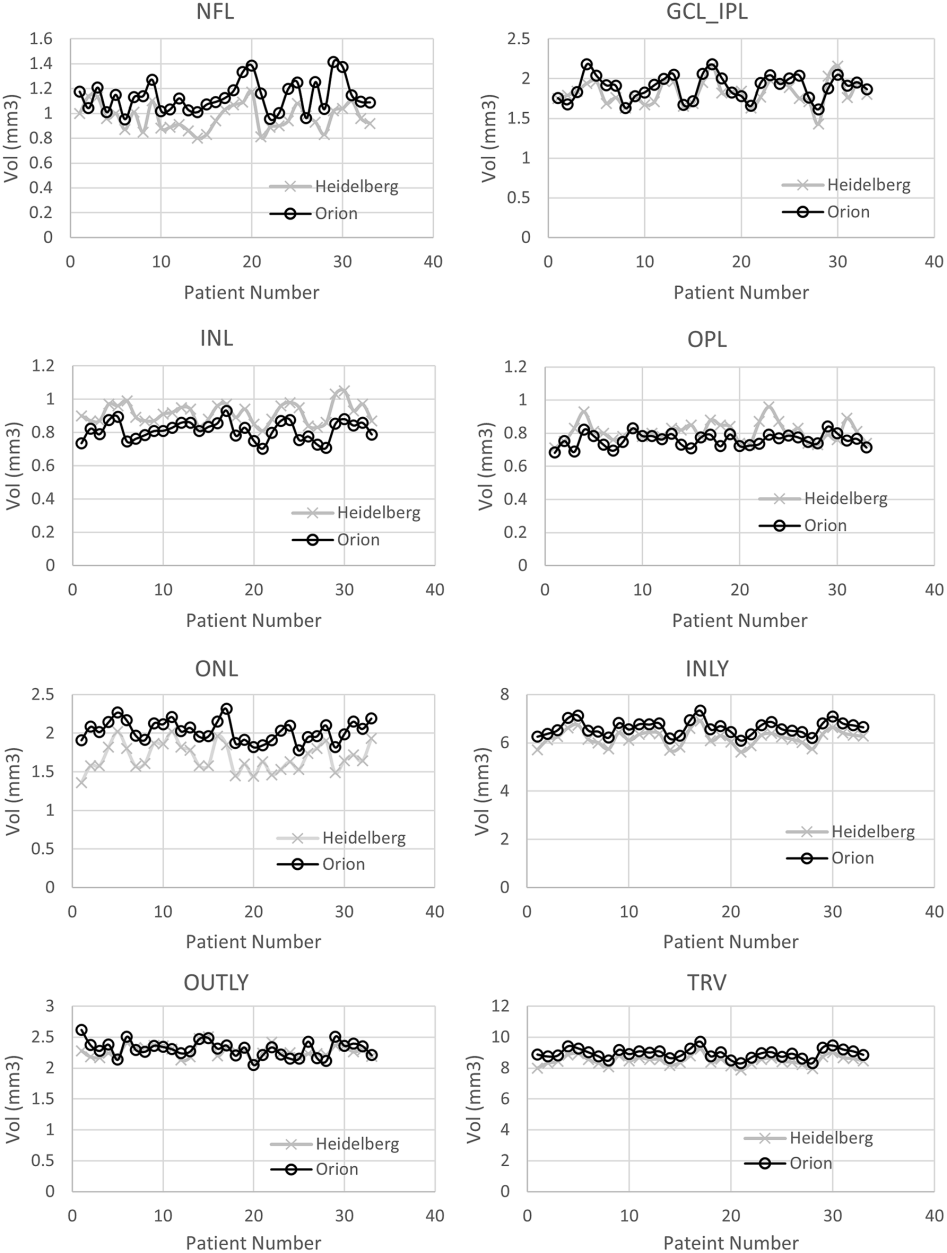
Comparison of retinal layer volumes between proprietary and cross-platform softwares in eyes with iAMD.

Using a quantitative analysis, the retinal layer segmentation volume was compared between the softwares in iAMD eyes to better understand differences in segmentation. The OPL layer volume measurement was found to have an extremely weak correlation between softwares. The NFL and OUTLY layers had a weakly positive correlation (r < 0.4), the GCL_IPL, INL and ONL layers had a moderate correlation (0.4 ≤ r < 0.7) while the INLY and TRV layers were strongly correlated (r > 0.7) between the two softwares (Table 4), (Fig. 3). Using a paired t-test, it was found that in GCL_IPL, OPL and OUTLY layers, there was no significant difference between the proprietary and the cross-platform softwares. In all other layers, the differences were statistically significant, p ≤ 0.05.

**Table 4:** Layer volume data in eyes with iAMD from the proprietary and the cross-platform softwares.

Vol. of the retinal layers in ETDRS zone (iAMD eyes)				
	Mean ± SD [proprietary software (Heidelberg)]	Mean ± SD [cross platform software (Orion)]	Pearson correlation	p value (paired t-test)
NFL	0.94 ± 0.11	1.12 ± 0.11	0.36	< 0.001
GCL_IPL	1.80 ± 0.16	1.85 ± 0.16	0.59	0.13
INL	0.94 ± 0.10	0.82 ± 0.05	0.50	< 0.001
OPL	0.82 ± 0.07	0.79 ± 0.05	0.04	0.17
ONL	1.69 ± 0.17	2.04 ± 0.12	0.56	< 0.001
INLY	6.18 ± 0.28	6.63 ± 0.28	0.78	< 0.001
OUTLY	2.34 ± 0.12	2.35 ± 0.11	0.35	0.59
TRV	8.52 ± 0.30	8.98 ± 0.31	0.69	< 0.001

### Retinal layer segmentations in eyes with DME

DME eyes were chosen to test retinal layer segmentation in eyes with inner retinal pathology. Thirty DME eyes were analyzed. On comparing both softwares to visual verification, 41.3% images were reported to have good segmentation, 25.3% were reported to have a mild error and 19.3% were reported as moderate segmentation error using the proprietary software while the cross-platform software reported 61.3% segmentation as good, 26% as mild error and 12.7% as moderate segmentation error (Table 1). A comparison performed using Wilcoxon test (Table 2) found that the cross-platform software was significantly better at segmenting GCIPL, INL and OPL layers (p ≤ 0.05). For other layers, there was no statistically significant difference. When comparing proprietary and the cross- platform software with visual grading in DME eyes, the average level of agreement between proprietary and cross- platform software for all layers was 37% and the kappa statistic average of all layers was 0.19. In eyes with DME, INL was the layer with most relative discrepancy in the qualitative analysis (Supplementary Table S1). For proprietary software images, the level of intergrader agreement for all layers was 53% with a kappa statistic average for all layers of 0.55 while for cross-platform images, the degree of intergrader agreement for all layers was 58% with a kappa statistic average of all layers of 0.30 (Supplementary Table S2). The test–retest kappa average for eyes with DME was 0.83.

**Figure 4:**
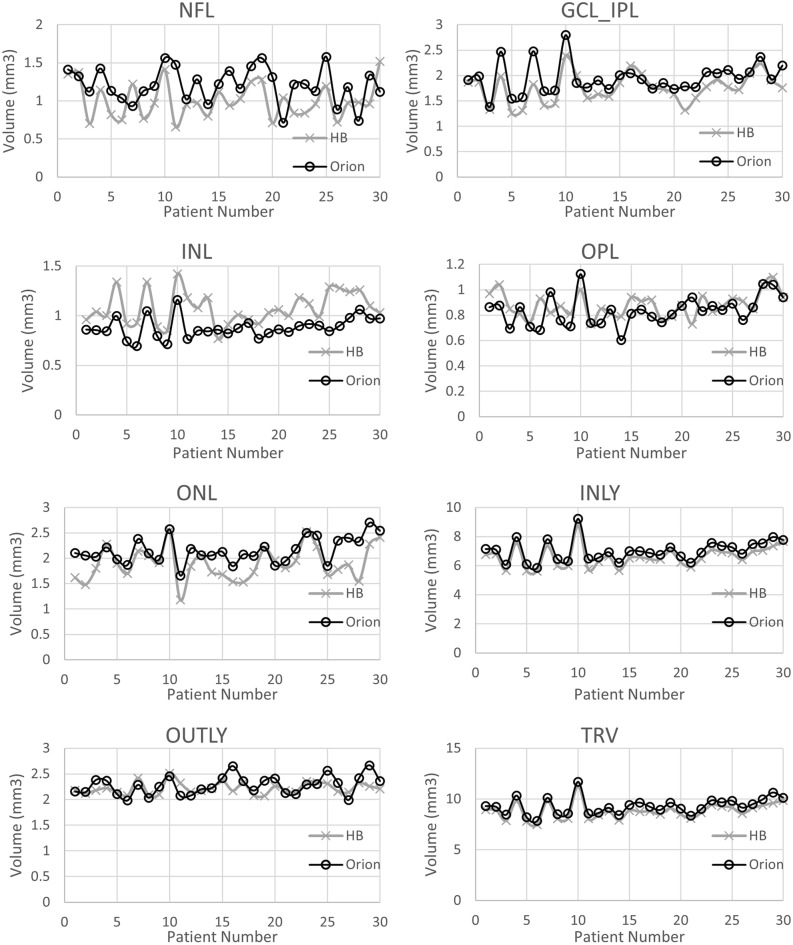
Comparison of retinal layer volumes between proprietary and cross-platform softwares in eyes with DME.

To better understand how the two softwares were different in segmenting DME eyes, a quantitative analysis was performed comparing retinal layer volumes after segmentation. The NFL, OPL and OUTLY layers showed moderate correlation (0.4 ≤ r < 0.7), while the GCL_IPL, INL, ONL, INLY and TRV layers showed strong correlation (r ≥ 0.7) between the proprietary and the cross-platform softwares (Table 5). Paired t-tests found a significant difference between all layer volumes except OPL and OUTLY layers (Table 5), (Fig. 4).

**Table 5:** Layer volume data in eyes with Diabetic Macular Edema (DME) from the proprietary and the cross-platform softwares.

Vol. of the retinal layers in ETDRS zone (DME eyes)				
	Mean ± SD [proprietary software (Heidelberg)]	Mean ± SD [cross platform software (Orion)]	Pearson Correlation	p value (paired t-test)
NFL	1.01 ± 0.23	1.21 ± 0.23	0.42	< 0.001
GCL_IPL	1.76 ± 0.28	1.95 ± 0.30	0.81	< 0.001
INL	1.08 ± 0.16	0.88 ± 0.10	0.70	< 0.001
OPL	0.88 ± 0.10	0.84 ± 0.12	0.48	0.04
ONL	1.90 ± 0.32	2.16 ± 0.25	0.73	< 0.001
INLY	6.62 ± 0.71	7.02 ± 0.72	0.99	< 0.001
OUTLY	2.22 ± 0.11	2.28 ± 0.18	0.43	0.07
TRV	8.83 ± 0.79	9.30 ± 0.82	0.99	< 0.000

### Comparison of old versus new updated viewing module of the Proprietary software (Heidelberg Spectralis)

The quantitative findings (layer volume values) from the image segmentation with the old viewing module (6.12.7.0) of the Spectralis software and the new viewing module (6.15.7.0), of the Spectralis software were compared in 10 eyes each of Normal, iAMD and DME, total of 30 eyes. The volume of each retinal layer analyzed by the new viewing module was found to be identical to the previous analysis by the old viewing module. For the same 30 eyes, a qualitative comparison was done by looking at the images with layer segmentation in the new viewing module versus the old one. The layer segmentation done by both the viewing module versions of the Spectralis software were found to be identical to each other. This conclusion was confirmed by the manufacturer (personal communication with Heidelberg Engineering).

### Comparison of old cross-platform software build versus new updated build

To attempt to ensure that there was no bias generated from slightly different line thicknesses or from graders being able to identify segmentation lines by color, a new build of the cross-platform software was kindly generated by the software developer on request as shown in Supplementary Figs. S1, S2 and S3. The new build did not change the algorithm for segmentation but just changed the line thickness to match that of the proprietary software as well as allowing the line colors to be changed to match that of the proprietary software. With this new cross-platform build, a sample subset of 30 images (10 from each group) was qualitatively reanalyzed to see if there was any difference in the qualitative grading of the new cross-platform images with reduced line thickness compared to the old one. The two masked graders, retina fellows (VA and TM) regraded the new build cross-platform images. The new cross-platform image gradings (obtained by comparing against the gold standard manual segmentation) were then qualitatively compared with the proprietary software gradings (obtained by comparing against the gold standard manual segmentation). Of all the images graded, none of the images had a grading by the two masked graders which differed by more than one quality category (good/mild/moderate/severe). In the cases where there was a difference in grading by the two graders, the difference was by only one quality category (good vs mild, mild vs moderate, moderate vs severe). In these instances of disagreement, a senior retinal specialist (SB) made a final independent decisive grading.

To statistically analyze this subset, we used the degree of intergrader agreement for all layers in Normal eyes, eyes with iAMD and DME eyes. This was done with both the original build and new updated build of the cross-platform software. For the original build of the cross-platform software, the overall level of observed intergrader agreement, in grading the images for all the three groups of eyes together was 73%, with a kappa statistic average of all layers of 0.39. For the updated new build of the cross-platform software, the overall level of observed intergrader agreement, in grading the images for all the three groups of eyes together was 70%, with a kappa statistic average of all layers of 0.49. Qualitative comparison (Wilcoxon test) of retinal segmentation grading using cross-platform and proprietary softwares was performed for a subset of patients. This was done in two ways. The first case used visual verification method for grading automated segmentation from both softwares and used the original build of the cross-platform software. The second case used true manual segmentation method for grading automated segmentation from both softwares and used the new cross-platform software build. Average score was calculated for all layers together using a grading scheme (Good = 1, Mild = 2, Moderate = 3 and Severe = 4). In the first case, the grading yielded an average score of 1.62 ± 0.95 for the proprietary software and 1.34 ± 0.72 for the cross-platform software, with Wilcoxon signed rank test p-value of 0.06. In the second case, with true manual segmentation and new cross-platform software build, the average scores were 1.60 ± 0.95 for proprietary software and 1.46 ± 0.79 for cross-platform software, with Wilcoxon signed rank test p-value of 0.25.

## Discussion

The aim of the present study was to compare automated retinal segmentation to manual segmentation using the third-party cross-platform software and the proprietary software in normal and diseased eyes, using images captured by the Heidelberg HRA + OCT machine. In addition, we also aimed to understand how the different softwares, segmented layers differently by analyzing them quantitatively.

We compared the two automated retinal layer segmentation softwares to manual grading using two expert graders. This method of validation has been used frequently to assess intraretinal segmentation software^16–20^. We used a modified grading system utilizing reference images to grade how deviated the automated segmentation was from the actual visual verification. Visual verification was performed first by two masked graders followed by a senior grader casting an independent decisive grading in cases where there was disagreement between the two graders. We also performed ‘gold standard’ manual segmentation in a subset of patient eyes by manually outlining each retinal layer and then compared it against the automated segmentation gradings of the proprietary and cross-platform softwares for a complete qualitative analysis. The cross-platform software has previously been compared to manual grading in normal retina using images acquired by the Heidelberg HRA + OCT machine^17^. In this previous study, 24 volume scans were both automatically segmented into 7 retinal layers and manually segmented by two experts. The study found that the mean differences and ranges between the software and manual raters were all within the axial resolution (~ 5 µm) of the device. We similarly found that the cross-platform software grading agreed well with manual grading as the former was found to have 97.3% good, 2.2% mild and 0.4% moderate segmentation error in normal eyes.

Several research softwares are now available for cross-platform segmentation of the retina^21–23^. A previous study tried comparing five automated intra-retinal segmentation softwares, which did not include the cross-platform software by Voxeleron LLC, Pleasanton, CA, USA, version 3.0.0. This previous study used 610 B-scans with a size of 768 × 496 pixels from only 10 eyes of mild non-proliferative diabetic retinopathy patients. The software compared included Heidelberg Spectralis (version 6), IOWA Reference Algorithm, Automated Retinal Analysis tools (AURA), Dufour’s Algorithm, OCTRIM3D^1,2^. The ‘ground truth’ was set as manual grading from macular SD-OCT volume data. Two experienced graders labeled 5 retinal surfaces in representative images of the SD-OCT volume dataset. The inter-observer differences were used to investigate the accuracy of software. Therefore, a total of 250 (5 surfaces per B-scan) were checked and manually outlined by each grader in the pathologic dataset. The inner retinal layers appear to be well delineated using the Heidelberg Engineering and IOWA software in normal human retina^2^. The softwares were compared for the capability to detect the different layer surfaces, the accuracy of segmentation, as well as the presence and ease of use of the input and output formats of the image data and segmented layers.

Other research software has previously looked and found changes in inner retinal layers in early AMD^24^. However, no comparison was made to manual grading and additionally no validation was performed in disease. Another study compared intraretinal segmentation of images obtained by Zeiss Cirrus HD-OCT machine (Carl Zeiss Meditec, Inc., Dublin, CA). The study compared segmentation by custom software and the Iowa Reference Algorithm OCT-Explorer (version 3.5) with 2 manual graders in Normal, early/intermediate AMD and advanced AMD eyes^25^. A third masked grader was used to grade the images using a 4-point ordinal score, similar to the one used in the present study, and found that both the new software and the Iowa Reference Algorithm OCT-Explorer performed intraretinal segmentation well in Normal and early/intermediate AMD eyes, but the accuracy dropped off in advanced AMD. Interestingly, the new algorithm performed better, although not significantly, than both manual graders in advanced disease as judged by the third masked grader. This was thought to be due to the relatively limited normal images used to train the Iowa Reference Algorithm. This again highlights the importance of using more real-world clinical data to enable the algorithms to correctly segment OCT images in diseased retina.

This paper is the first to compare intraretinal segmentation of cross-platform OCT layer segmentation software in diseased retina to manual segmentation. We found that the proprietary and the cross-platform software grading agreed well with manual grading in eyes with normal retina but the agreement in retinal segmentation fell to 79% good, 15.8% mild and 4.9% moderate segmentation error in eyes with outer retinal pathology and fell even further to 61.3% good, 26% mild and 12.7% moderate segmentation error in eyes with intra-retinal pathology when the cross-platform software was compared with manual grading. We also found that the cross-platform software was subjectively better at segmenting retinal layers than the proprietary software in both normal and particularly in diseased eyes. Part of the difference between the proprietary and the cross-platform software stems from the difference in layer naming convention followed by the two softwares. The proprietary software follows the International Nomenclature for the classification of retinal layers on OCT images whereas the third-party cross-platform software uses the APOSTEL recommendation^26,27^ (Supplementary Fig. S4). Difference in algorithms used for the segmentation by the two softwares is difficult to estimate as exact details of the algorithms are not disclosed by the vendors.

**Figure 5:**
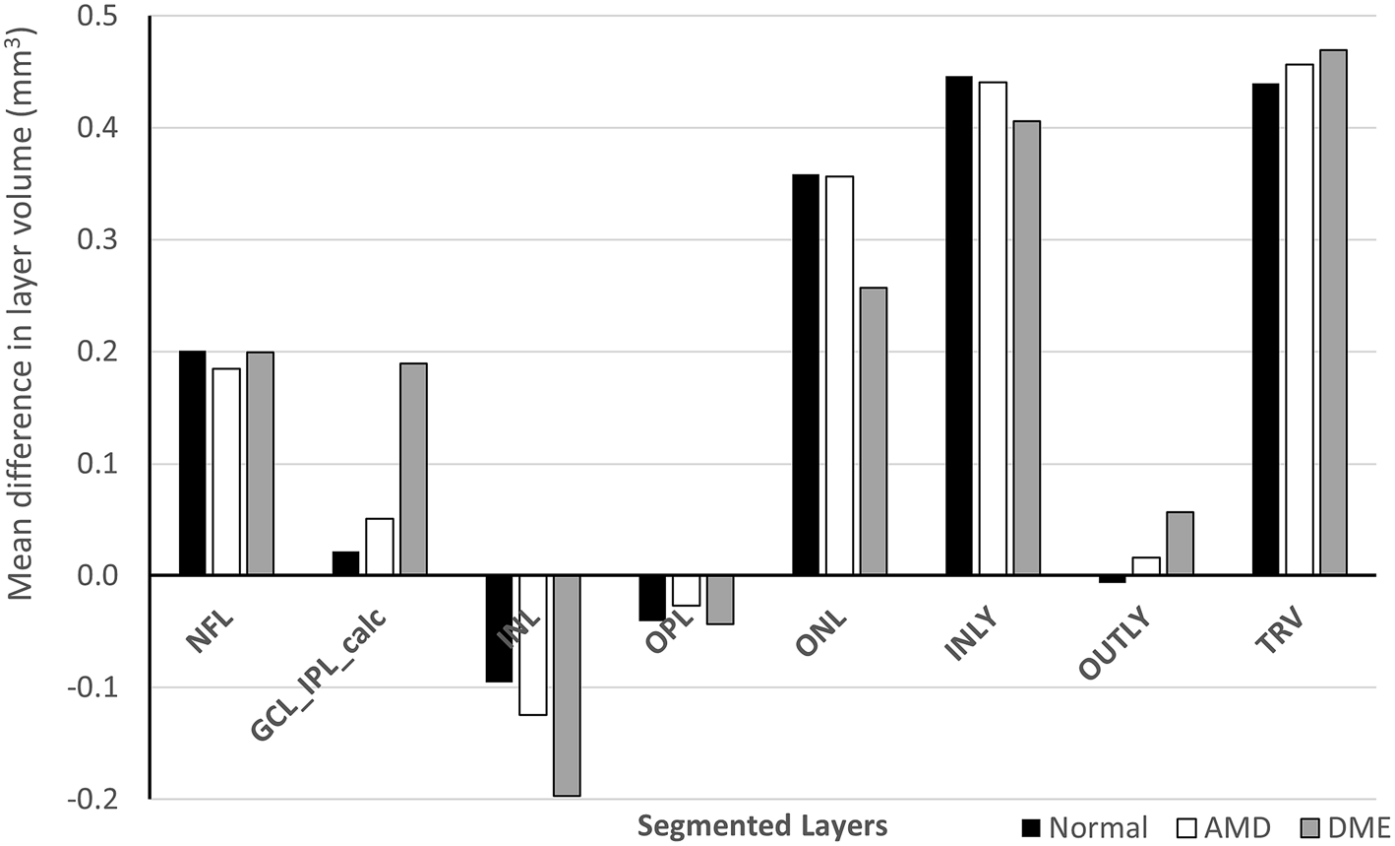
Mean difference between layer volumes measured by the proprietary and the cross-platform softwares expressed in mm^3^ for different types of patients across different segmented layers.

In terms of the differences in the final segmentation results of the two softwares, we found that the retinal layer segmentations correlated well between the two advanced softwares. However, in general, the layer volumes were significantly different. ONL was the layer with maximum difference (in measured layer volume) between the proprietary and the cross-platform software in both normal and diseased eyes. Inner and outer retinal layers and the total retinal volume also matched very well (within 0 to 10%) between the two softwares for all types of eyes, normal and with pathology, as shown in Fig. 5, Supplementary Figs. S5, S6 & S7. In Normal eyes, the layers GCL_IPL, INL and OPL matched very well (within 1 to 10%) between the proprietary and the cross-platform softwares. NFL and ONL layers matched moderately well (within 10 to 20%). In iAMD eyes for retinal segmentations, the GCL_IPL and OPL layers matched very well (within 1 to 10%) between the two softwares while NFL, INL and ONL layers matched moderately well (within 10 to 20%). In DME eyes for retinal segmentations, the GCL_IPL and OPL layers matched very well (within 1 to 10%) between the two softwares while NFL, INL and ONL layers matched moderately well (within 10 to 20%) as shown in Fig. 5. In the eyes with DME, ONL layer had the maximum difference in measured layer volume between the proprietary and the cross-platform software. In terms of percentage difference of measured layer volume in DME eyes, INL layer had the maximum difference between the softwares.

The cross-platform software has been used previously in cross- platform comparisons^28^. Zeiss Cirrus and Heidelberg HRA + OCT images were compared using the cross-platform software but only for retinal segmentation. Using the said software, there was good compatibility of total retinal thickness measurements when analyzing images from the same subject using both devices. In the present study, differences in the retinal layer segmentation between the two automated softwares is likely due to differences in the method used by the algorithm to segment the retina. We requested access to the algorithm to better understand the differences between the softwares, but this was not provided due to protection of intellectual property (private communications). Research groups have used a variety of algorithms to perform intra-retinal segmentation including using graph-based^29–32^, active contour^33^ and texture models^34^.

One of the limitations of our study was that only the horizontal B scans crossing the fovea were tested, as it was felt that this was the most common scan used by physicians in clinic. The findings of comparison may have been different in other areas of the macula beyond the fovea. Another limitation of making conclusions about which software might be better for images likely to be found in the clinical setting was that we used only DME retina to represent inner retinal pathology and iAMD to represent outer retinal pathology. In the clinical setting, there is a far larger variety of retinal pathologies. Future studies should ideally look at comparing automated intra-retinal segmentation in a wider spectrum of retinal diseases to provide a better representation of how the software performs in the real-world clinic setting.

In summary, this paper adds to the knowledge regarding how different software platforms perform intra-retinal segmentation in normal and in diseased eyes. We found that although the proprietary and the cross-platform software measurements of retinal layer volumes were correlated, they were found to be significantly different suggesting that different retinal landmarks were chosen for identifying retinal layers. Both softwares performed well in segmenting normal and diseased retina, when compared with manual segmentation/visual verification, the cross-platform software being subjectively better than the proprietary software. However, our findings suggest caution in using the software for intra-retinal segmentation in diseased eyes as the segmentation errors increase in pathology when compared to normal retina, particularly so in the proprietary software. Thus, the cross-platform software offers a reliable alternative for intra-retinal segmentation which may be particularly useful for research studies and clinical trials as its clinical use is not yet approved.

## Supplementary Information

Supplementary Information.

## Abbreviations

HB: Heidelberg
iAMD: Intermediate dry age-related macular degeneration
DME: Diabetic macular edema
OR: Orion
TRV: Total retinal volume
GCIPL: GCL + IPL
OUTLY: Outer retinal layer in toto
INLY: Inner retinal layer in toto
