# Supplementary material for: Assessing the validity of a cross-platform retinal image segmentation tool in normal and diseased retina
Source: Sci Rep. 2021 Nov 8;11:21784. doi: 10.1038/s41598-021-01105-9 (PMC8575997; doi:10.1038/s41598-021-01105-9)
Supplement: Supplementary file 1 — Supplementary Information. [file 41598_2021_1105_MOESM1_ESM.pdf]

SUPPLEMENTARY DATA

Assessing the validity of a cross-platform retinal image segmentation tool in normal and diseased retina

Varsha Alex[1], Tahmineh Motevasseli[1], William R Freeman[1], Jefy A Jayamon[2], Dirk-Uwe G. Bartsch[1], Shyamanga Borooah[1*]

Affiliations:
[1] Jacobs retina center, Shiley Eye Institute, UCSD, USA
[2] Qualcomm Technologies Inc, San Diego, California, USA

Supplementary Figure S1

Retinal layer segmentation of Normal eyes using the proprietary and the cross-platform software.

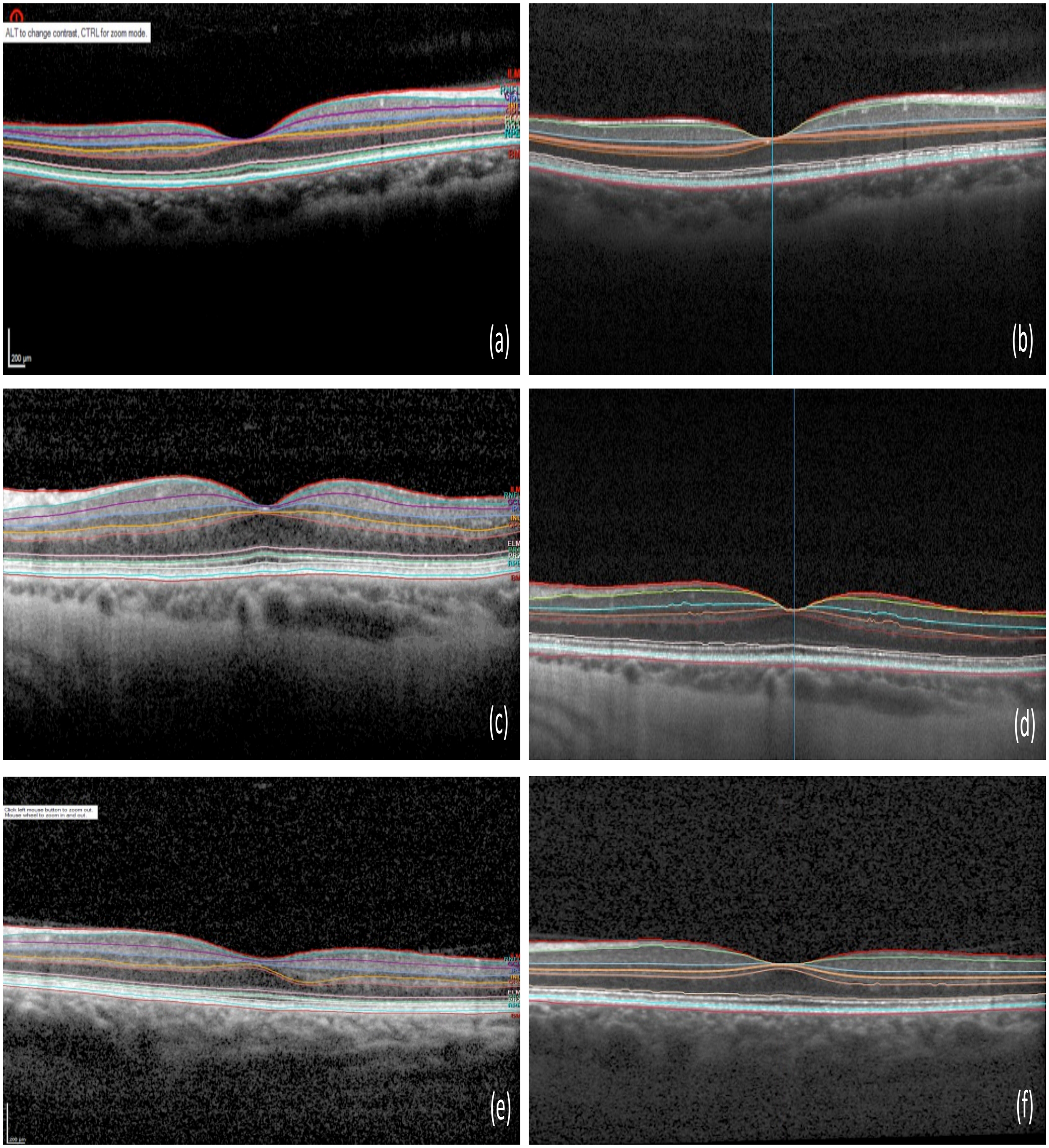

Figure 1. (a) Heidelberg with no errors (b) Orion with no errors (c) Heidelberg with mild errors (d) Orion with mild errors (e) Heidelberg with moderate segmentation errors (f) Orion with moderate segmentation errors.

Supplementary Figure S2

Retinal layer segmentation of eyes with iAMD using the proprietary and the cross-platform software

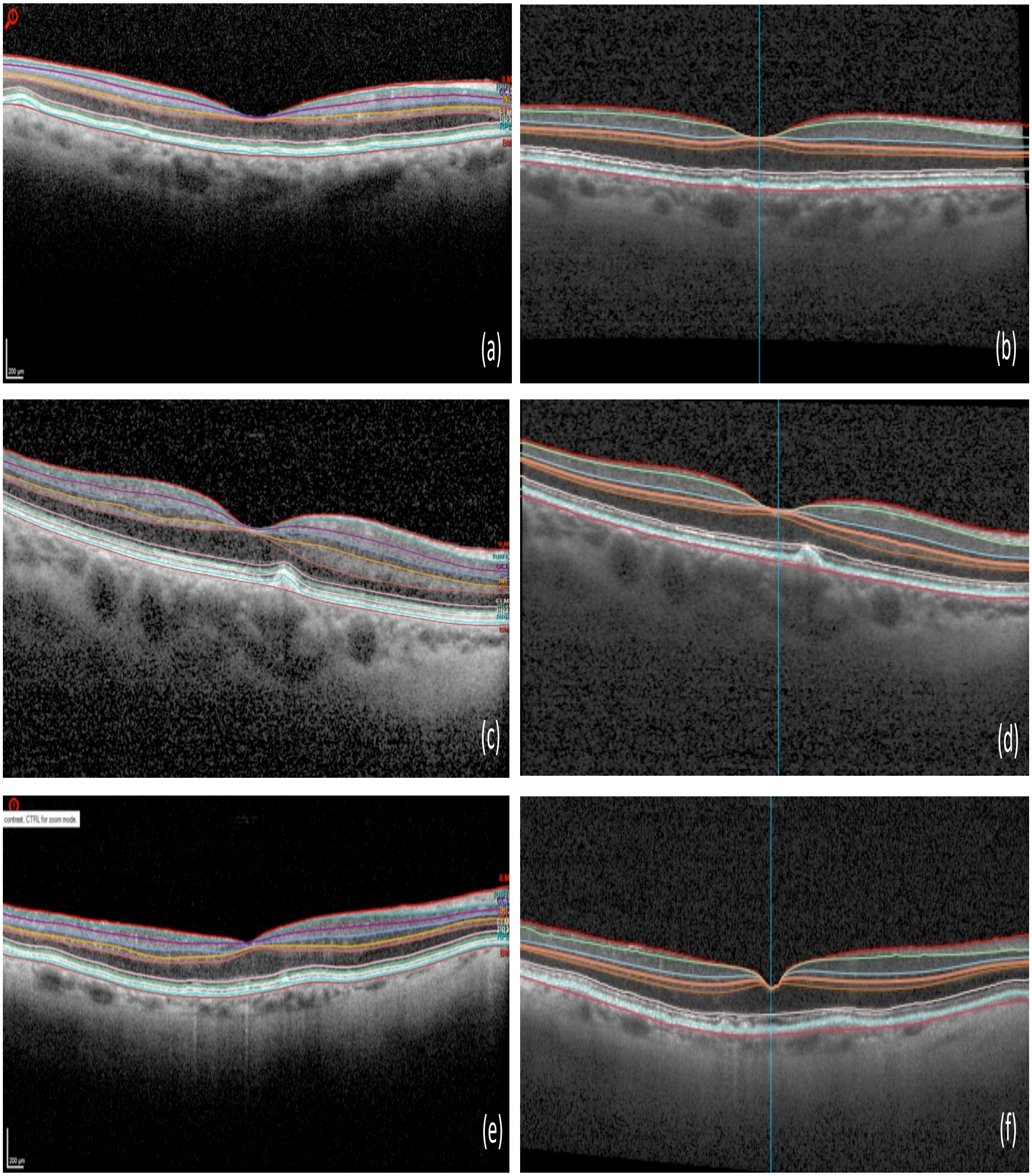

Figure 2. (a) Heidelberg with no errors (b) Orion with no errors (c) Heidelberg with mild errors (d) Orion with mild errors (e) Heidelberg with moderate segmentation errors (f) Orion with moderate segmentation errors.

Supplementary Figure S3

Retinal layer segmentation of DME eyes using the proprietary and the cross-platform software

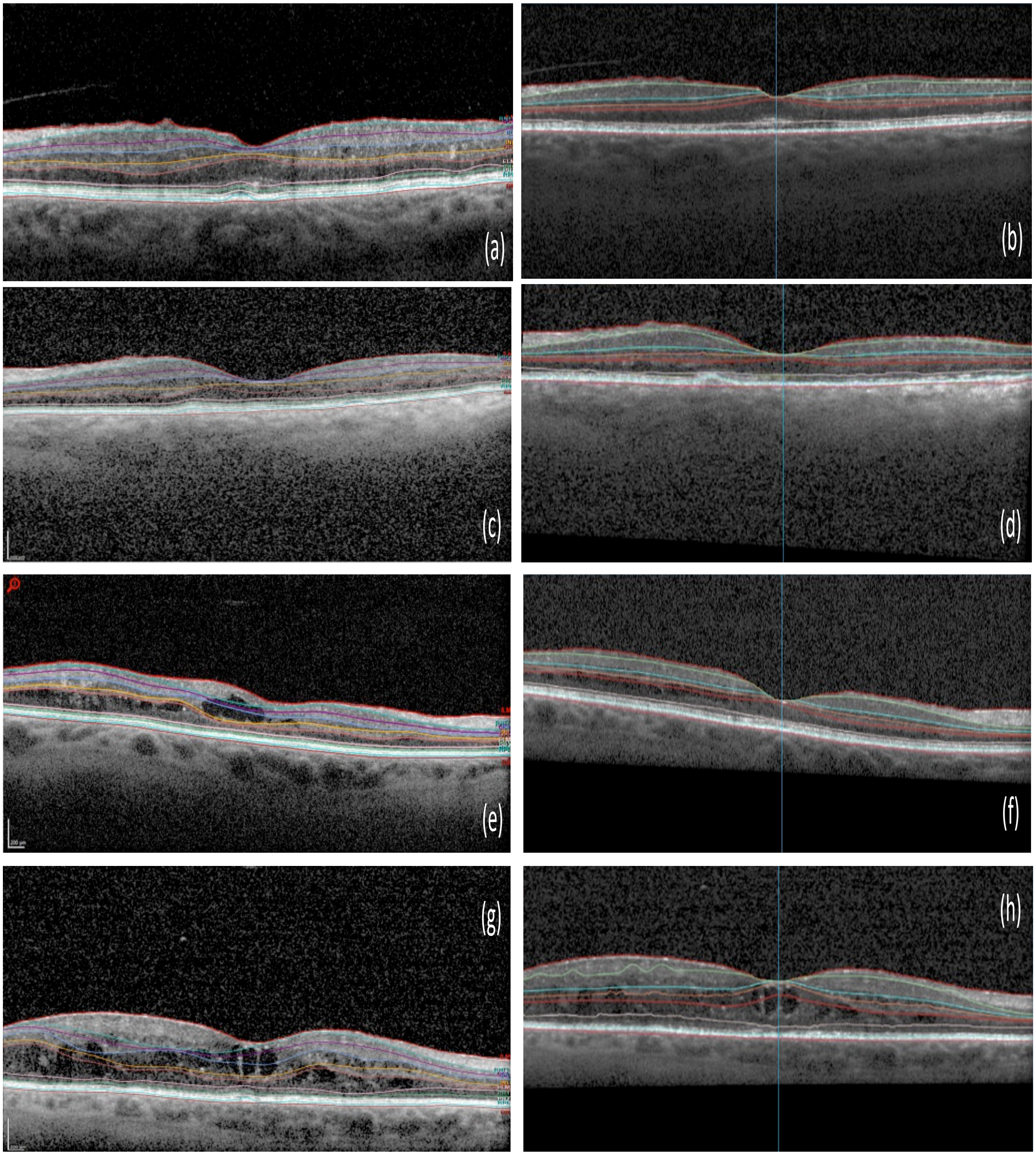

.

Figure 3. (a) Heidelberg with no errors (b) Orion with no errors (c) Heidelberg with mild errors (d) Orion with mild errors (e) Heidelberg with moderate segmentation errors (f) Orion with moderate segmentation errors (g) Heidelberg with severe segmentation errors (h) Orion with severe segmentation errors.

Supplementary Figure S4

International Nomenclature

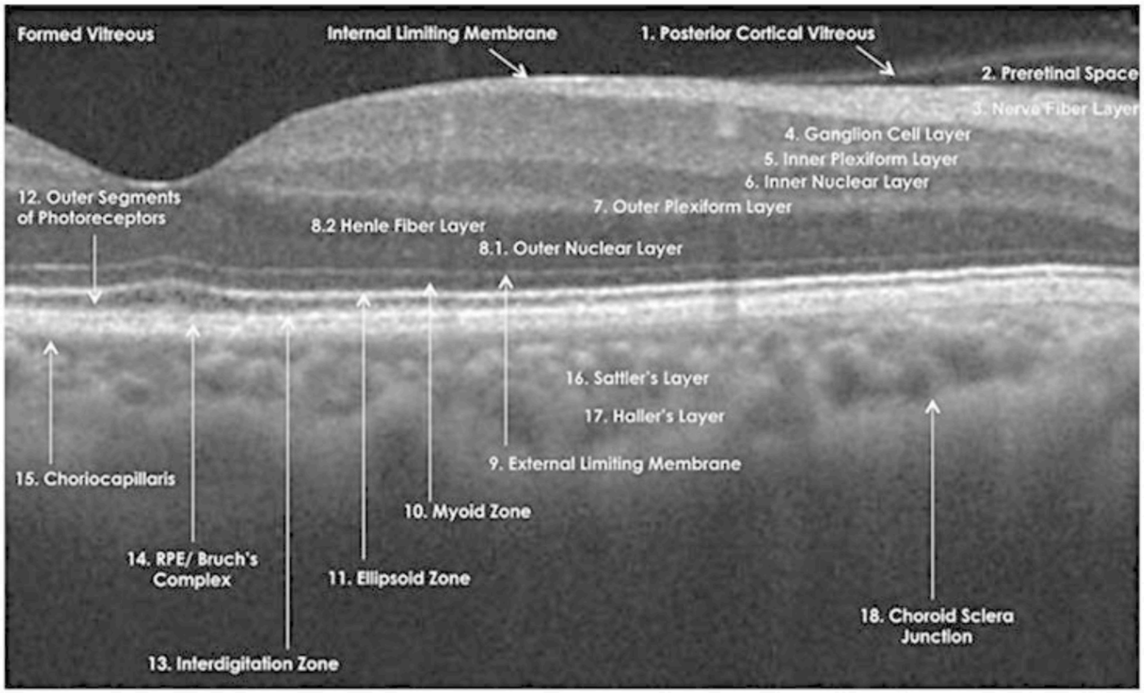

APOSTEL recommendation

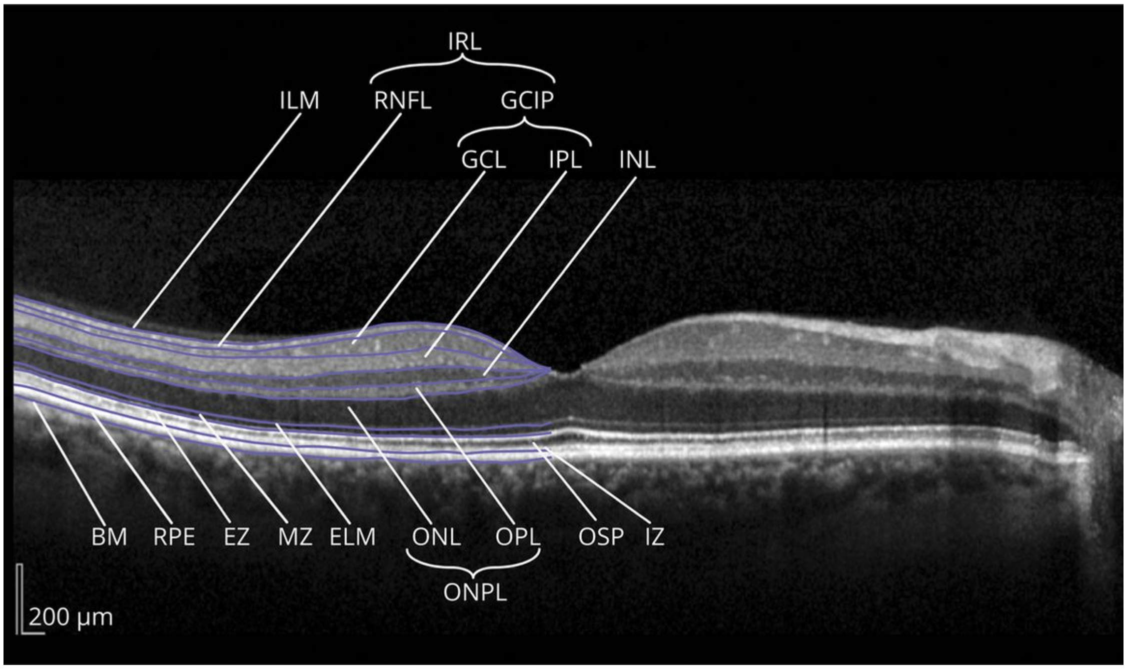

Supplementary Figure S5

Comparison of segmented retinal layer volumes in Normal eyes using the proprietary and the cross-platform software

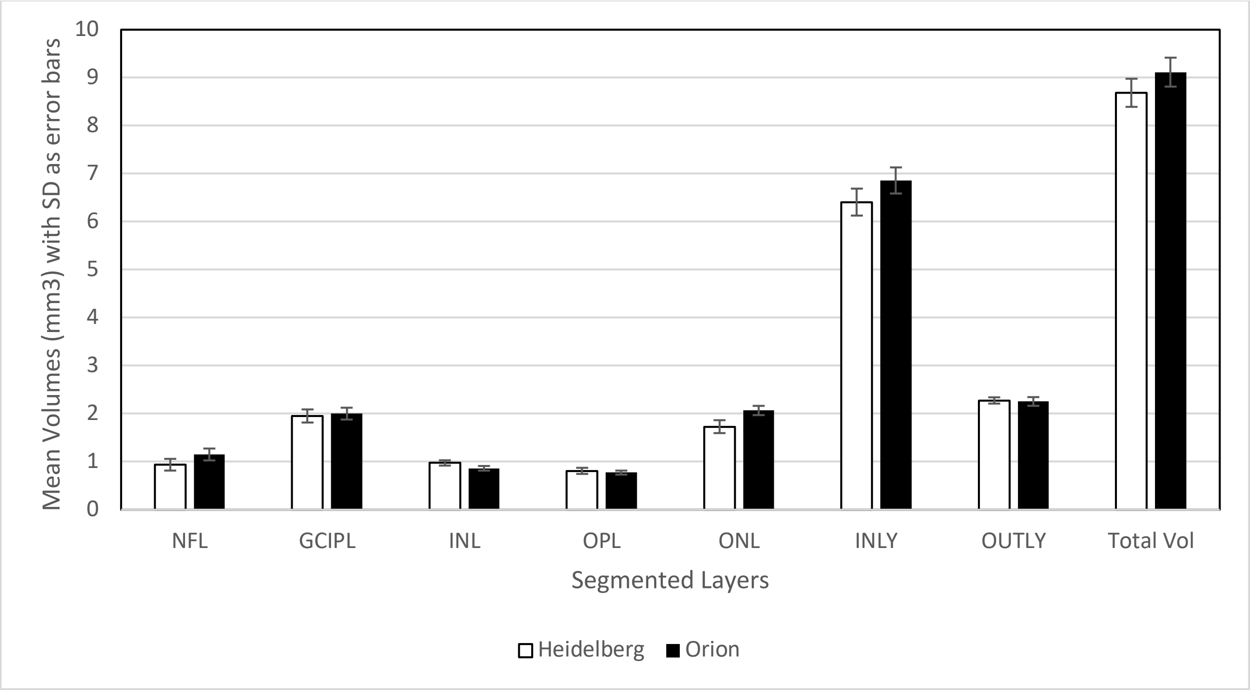

INLY- Inner retinal layers in total, OUTLY- Outer retinal layers in total

Supplementary Figure S6

Comparison of segmented retinal layer volumes in intermediate dry age-related macular degeneration eyes using the proprietary and the cross-platform software

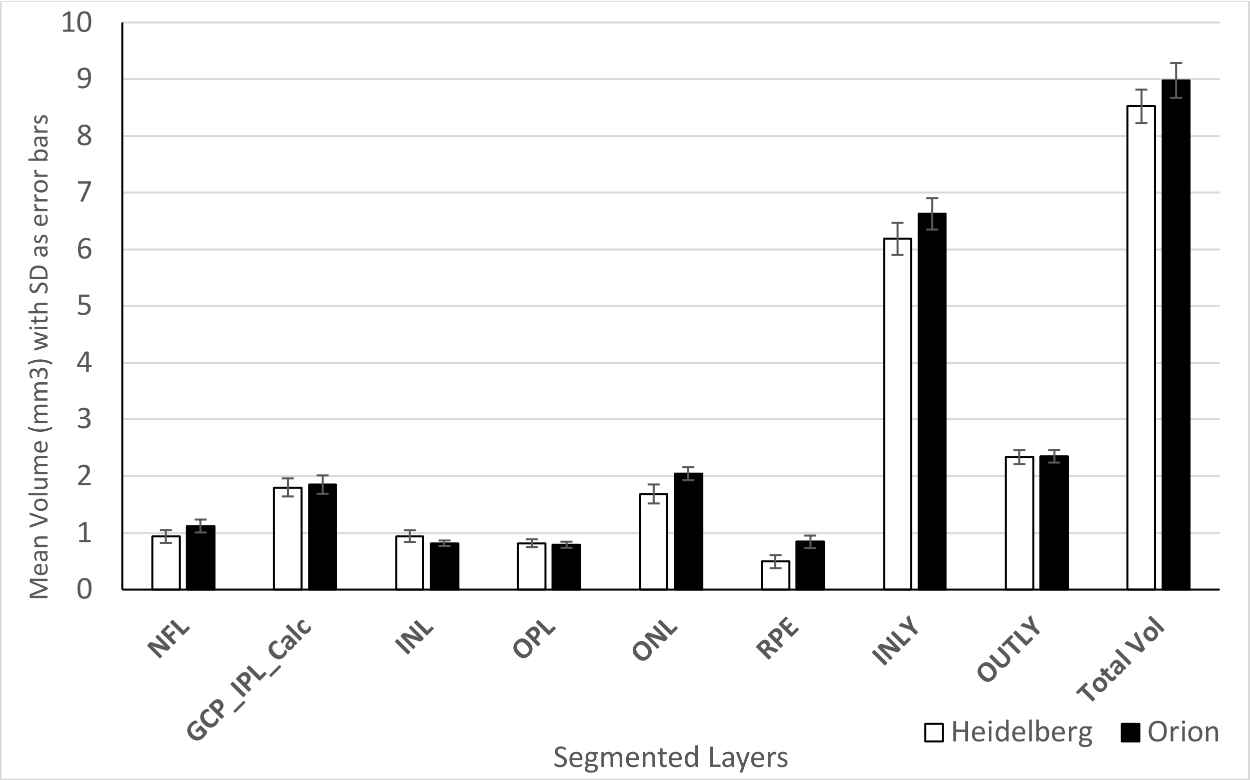

INLY- Inner retinal layers in total, OUTLY- Outer retinal layers in total

Supplementary Figure S7

Comparison of segmented retinal layer volumes in eyes with diabetic macular edema using the proprietary and the cross-platform software

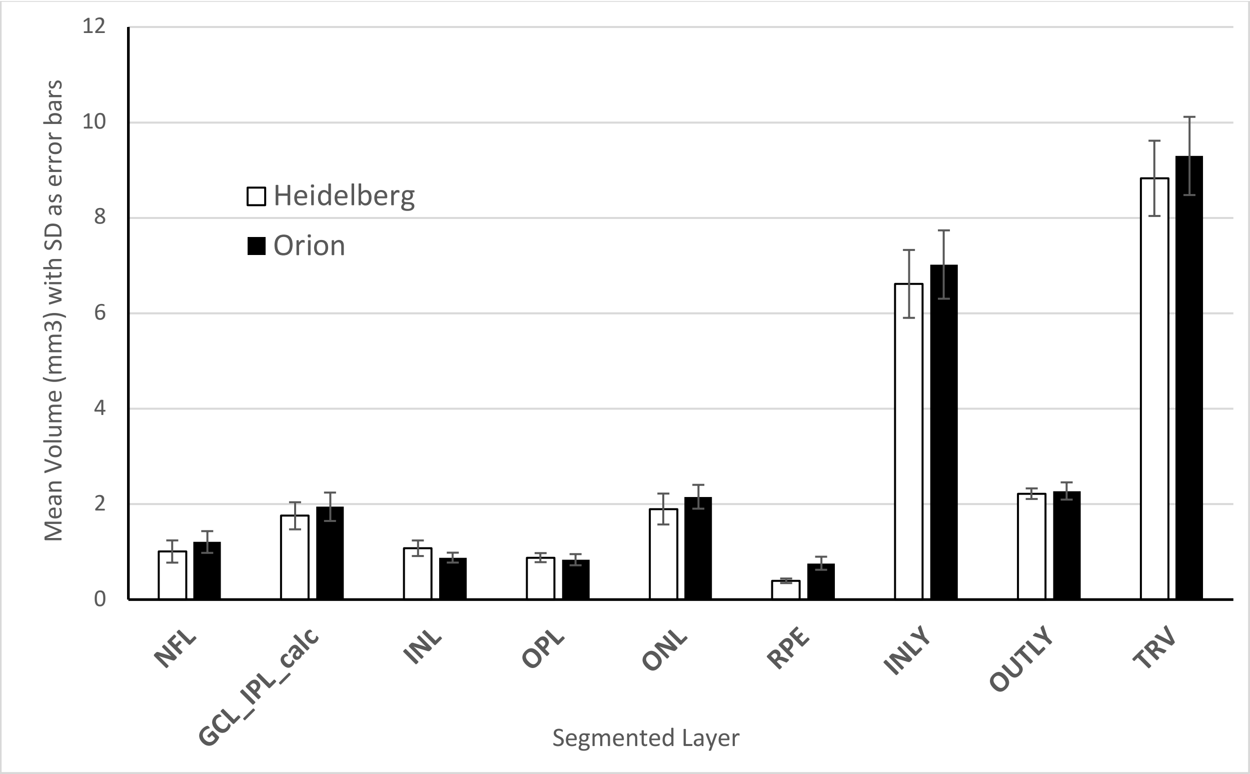

TRV- Total retinal volume, INLY- Inner retinal layers in total, OUTLY- Outer retinal layers in total

Supplementary Table 1

Qualitative comparison of the proprietary and the cross-platform software retinal segmentations, with manual grading, in normal, intermediate dry age-related macular degeneration and diabetic macular edema eyes

**Normal**		
**Layer**	**Observed Agreement**	**Weighted Kappa**
NFL	0.53	0.32
GCIPL	0.82	0.31
INL	0.80	0.33
OPL	0.91	0.33
ONL	0.91	0.30
Average	0.80	0.32
**AMD**		
**Layer**	**Observed Agreement**	**Weighted Kappa**
NFL	0.73	0.40
GCIPL	0.76	0.13
INL	0.73	0.36
OPL	0.85	0.59
ONL	0.58	0.32
Average	0.73	0.36
**DME**		
**Layer**	**Observed Agreement**	**Weighted Kappa**
NFL	0.43	0.25
GCIPL	0.47	0.19
INL	0.23	0.13
OPL	0.33	0.18
ONL	0.40	0.21
Average	0.37	0.19

Supplementary Table 2

Intergrader agreement in Normal, Intermediate dry AMD and DME eyes

NORMAL - HEIDELBERG		
**Layer**	**Observed Agreement**	**Weighted Kappa**
NFL	0.76	0.33
GCIPL	0.58	0.31
INL	0.73	0.34
OPL	0.84	0.31
ONL	0.87	0.33
Average	0.76	0.32
**NORMAL - ORION**		
**Layer**	**Observed Agreement**	**Weighted Kappa**
NFL	0.91	0.54
GCIPL	0.93	0.33
INL	0.98	0.33
OPL	0.76	0.33
ONL	0.91	0.33
Average	0.90	0.38
**AMD - HEIDELBERG**		
**Layer**	**Observed Agreement**	**Weighted Kappa**
NFL	0.39	0.12
GCIPL	0.70	0.32
INL	0.41	0.27
OPL	0.61	0.26
ONL	0.70	0.50
Average	0.56	0.29

AMD - ORION		
**Layer**	**Observed Agreement**	**Weighted Kappa**
NFL	0.61	0.27
GCIPL	0.88	0.73
INL	0.85	0.56
OPL	0.70	0.35
ONL	0.69	0.61
Average	0.74	0.51
**DME - HEIDELBERG**		
**Layer**	**Observed Agreement**	**Weighted Kappa**
NFL	0.63	0.66
GCIPL	0.60	0.58
INL	0.70	0.68
OPL	0.43	0.46
ONL	0.30	0.38
Average	0.53	0.55
**DME - ORION**		
**Layer**	**Observed Agreement**	**Weighted Kappa**
NFL	0.67	0.50
GCIPL	0.73	0.54
INL	0.63	0.21
OPL	0.46	0.09
ONL	0.40	0.19
Average	0.58	0.30

Supplementary Table 3

Summary of comparison of two automated segmentation softwares

	Proprietary software	Cross-platform software
1. Disclaimer	Not good for pathology segmentation	Not approved for clinical use
2. Layer convention	International Nomenclature	APOSTEL recommendation
3. EDI images	Cannot segment	Can segment
4. Images analyzable	Proprietary/Integrated	Cross platform
